# Evaluation of Nutritional Status in Hospitalized Chronic Obstructive Pulmonary Disease Patients and Can C-reactive Protein-to-Albumin Ratio Be Used in the Nutritional Risk Assessment in These Patients

**DOI:** 10.7759/cureus.21833

**Published:** 2022-02-02

**Authors:** Ramazan Baldemir, Ayperi Öztürk, Guler Eraslan Doganay, Mustafa Ozgur Cirik, Ali Alagoz

**Affiliations:** 1 Anesthesiology and Reanimation, Ankara Atatürk Chest Diseases and Thoracic Surgery Training and Research Hospital, Ankara, TUR; 2 Chest Disease, Ankara Atatürk Chest Diseases and Thoracic Surgery Training and Research Hospital, Ankara, TUR; 3 Anesthesiology and Reanimation, Ankara Ataturk Chest Diseases and Thoracic Surgery Training and Research Hospital, Ankara, TUR

**Keywords:** nutritional risk score-2002, nutrition, c- reactive protein-to-albumin ratio, c- reactive protein, chronic obstructive pulmonary disease, albumin

## Abstract

Background

Malnutrition is common in patients diagnosed with chronic obstructive pulmonary disease (COPD). CRP/albumin ratio (CAR) can be used as a parameter to evaluate the inflammatory process and nutritional status together. The aim of this study was to make a general evaluation of the nutritional status of hospitalized patients with COPD and to investigate whether CAR can predict nutritional status in these patients.

Methods

Patients who were hospitalized with COPD who were consulted to the nutrition department were included in the study. The patients' Nutritional Risk Score-2002 (NRS), demographic data, diagnoses, body mass indexes (BMI), nutritional support applied to the patients were recorded. CRP, Albumin, and CAR values of the patients were determined. Patients recommended nutritional follow-up, total parenteral nutrition (TPN) or enteral nutrition (EN) initiated, and oral nutritional supplement (ONS) support were identified.

Results

A total of 393 patients with COPD were analyzed. 88.55% of the patients were in the NRS ≥ 3 risk group. TPN treatment was started in 10.2% of the patients, EN in 10.9%, ONS in 76.3%, and nutritional follow-up was recommended in 2.5% of the patients. While albumin level was lower in patients with NRS ≥ 3, CRP and CAR were higher in patients with NRS ≥ 3 (p < 0.05). There was a negative correlation between NRS-2002 and albumin (p < 0.001). A positive correlation was observed between NRS-2002 and CRP and CAR (p < 0.001). Age and CAR were found to be effective in predicting those with NRS-2002 ≥ 3. The cut-off value for CAR was accepted as 3.26.

Conclusions

The need for nutritional support is high in patients hospitalized with COPD. It is important to evaluate nutritional support needs in these patients, regardless of NRS-2002 and BMI. In this respect, the clinician's observation and the decision are as valuable as the scoring that determines malnutrition. We think that the cut-off value of 3.26 determined for CAR can be used in the nutritional risk assessment in patients with COPD.

## Introduction

It is known that malnutrition that is not managed well worsens the course of the primary disease, prolongs the hospital stay, and adversely affects complications [1]. Malnutrition is common in patients diagnosed with chronic obstructive pulmonary disease (COPD), and it is an important condition that negatively affects morbidity and mortality [2-4]. The chronic inflammatory process encountered in these patients negatively affects the nutritional status [5,6]. This may differ in the assessment of nutritional status in patients with COPD due to chronic inflammation. The European Society of Parenteral and Enteral Nutrition (ESPEN) also made a definition in 2016 by considering patients with and without systemic inflammation in two different malnutrition subcategories based on biochemical inflammatory markers [7]. COPD is an inflammatory condition of the respiratory system that causes airway obstruction, and it is known that there is an increase in serum inflammatory marker levels in patients with COPD [8-11]. However, the relationship between inflammatory markers and nutritional status in patients with COPD is an issue that needs to be investigated in the literature. Although there are many inflammatory markers used in practice, C-reactive protein (CRP) is generally used as a laboratory parameter in the evaluation of inflammatory status [12]. Albumin, on the other hand, is considered a nutritional parameter, although there is no consensus on it [13]. CRP-to-albumin ratio (CAR), calculated by CRP and albumin values, is used in the literature for prognosis evaluation in many diseases [12]. CAR can also be used as a parameter to evaluate the inflammatory process and nutritional status together. The aim of this study is to make a general evaluation of the nutritional status of hospitalized patients with COPD and to investigate whether CAR can predict nutritional status in COPD patients.

## Materials and methods

This retrospective study was performed following the approval of the Ethics Committee (Ankara Keçiören Training and Research Hospital, Date: 11.01.2022, Number: 2012-KAEK-15/2447). The records of the patients evaluated in the nutrition department for the period of January 2019 and November 2019 were examined. Patients who were hospitalized with COPD who were consulted to the nutrition department were included in the study.

The patients' Nutritional Risk Score-2002 (NRS), demographic data, diagnoses, body mass indexes (BMI), and nutritional support applied to the patients were recorded. CRP, albumin, and CAR values of the patients were determined. Patients recommended nutritional follow-up, total parenteral nutrition (TPN) or enteral nutrition (EN) initiated, and oral nutritional supplement (ONS) support were identified.

Patients in the intensive care unit, those with missing data, those using anti-inflammatory drugs, and those hospitalized for a reason other than COPD were not included in the study. CAR was calculated by dividing the CRP value by the albumin value. The patients were evaluated in four groups according to BMI; BMI < 18.5 kg/m2 (underweight), BMI: 18.5-24.9 kg/m2 (normal), BMI: 25-29.9 kg/m2 (overweight), BMI ≥ 30 kg/m2 (obese). According to NRS-2002, patients were evaluated in two groups. NRS-2002; one and two, NRS-2002; ≥ 3.

Statistical analysis

Data analyses were performed by using Statistical Package for the Social Sciences [(SPSS) (IBM Corp. Released 2013. IBM SPSS Statistics for Windows, Version 22.0. Armonk, NY: IBM Corp)]. Whether the distribution of continuous variables was normal or not determined by the Kolmogorov Smirnov test. Levene test was used for the evaluation of homogeneity of variances. Unless specified otherwise, continuous data were described as mean ± standard deviation (SD) for normal distributions, and median (inter-quartile range) for skewed distributions. Categorical data were described as the number of cases (%). Statistical analysis differences in not normally distributed variables between two independent groups were compared by the Mann Whitney U test. Statistical analysis differences in not normally distributed variables between four independent groups were compared by the Kruskal Wallis test. Categorical variables were compared using Pearson’s Chi-square test or Fisher’s exact test. Univariate and multivariate binary logistic regression analyses were performed to assess the association between mortality and the risk factors findings. Receiver operating characteristic (ROC) curve analysis was used to determine the cut-off value of the albumin, CRP ve CRP/albumin ratio associated with the risk of NRS 2002 score. It was evaluated the degrees of the relationship between variables with Spearman correlation analysis. It was accepted p-value < 0.05 as a significant level on all statistical analyses.

## Results

**Figure 1:**
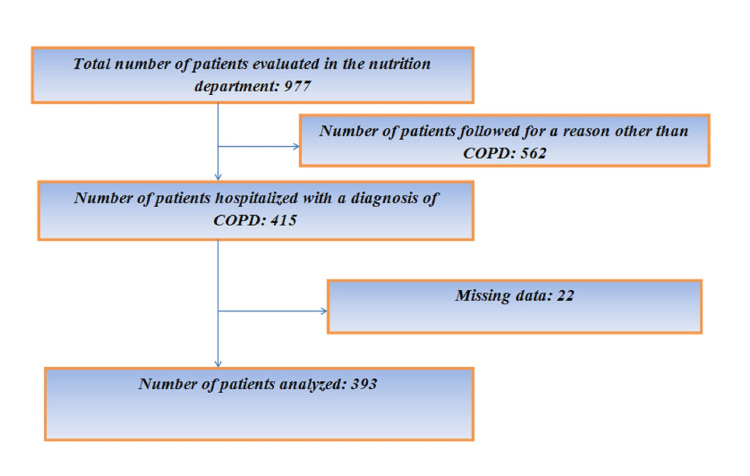
Flow chart of the patients COPD: chronic obstructive pulmonary disease

Of the 977 patients evaluated in the nutrition department, 42.4% (415 patients) were hospitalized due to COPD. Twenty-two of these patients were excluded from the study due to missing data. A total of 393 patients with COPD were analyzed (Figure 1).

Demographic data, comorbidities, and NRS-2002 scores of the patients are given in Table 1.

**Table 1:** Demographic data, comorbidities, and NRS-2002 scores of the patient Continuous variables are expressed as either the mean ± Standard deviation (SD) and categorical variables are expressed as either frequency (percentage). BMI: Body mass index, NRS: Nutritional risk score

	All Patients (n:393)
AGE, years, mean ± SD	68.99 ± 13.06
Gender, n(%)	
Male	279 (71.0%)
Female	114 (29.0%)
BMI (kg/m²), mean ± SD	23.39 ± 4.91
Comorbidities, n(%)	
Pneumonia	86 (21.9%)
Pulmonary thromboembolism	12 (3.1%)
Diabetes mellitus	34 (8.7%)
High blood pressure	32 (8.1%)
Heart disease	38 (9.7%)
Kidney failure	9 (2.3%
Alzheimer	9 (2.3%)
Hypothyroidism	8 (2.0%)
NRS-2002=1 or 2	45(11.45%)
NRS-2002 ≥ 3	348(88.55%)

TPN treatment was started in 10.2% of the patients, EN in 10.9%, ONS in 76.3%, and nutritional follow-up was recommended in 2.5% of the patients without any nutritional treatment.

When we evaluated the relationship between the nutritional treatments applied to the patients and the BMI of the patients, there was no statistically significant difference between the nutritional treatments (TPN, EN, ONS) applied according to BMI (p > 0.05).

When the nutritional application results of the patients were compared according to the NRS -2002 groups, a statistically significant difference was found only in terms of food follow-up (p < 0.05). Nutrition follow-up was statistically significantly lower in those with NRS ≥ 3 compared to those with NRS one and two (p < 0.05) (Table 2).

**Table 2:** Nutrition Applications of the Patients According to NRS-2002 ONS: Oral Nutritional Supplement, TPN: Total Parenteral Nutrition, EN:  Enteral nutrition, NRS: Nutritional Risk Score

	NRS 1 or 2 (n:45)	NRS ≥3 (n:348)	P
	n (%)	n (%)	
TPN	1 (2.2%)	39 (11.2%)	0.067
ONS	33 (73.3%)	267 (76.7%)	0.615
EN	5 (11.1%)	38 (10.9%)	0.999
Nutrition follow-up	6 (13.3%)	4 (1.1%)	<0.001

When the patients were compared in terms of laboratory values according to the NRS-2002 groups, a statistically significant difference was observed in terms of albumin, CRP, and CAR values compared to the NRS-2002 groups (p < 0.05). While albumin was statistically significantly lower in patients with NRS-2002 ≥ 3, CRP and CAR were statistically significantly higher in patients with NRS-2002 ≥ 3 (p < 0.05) (Table 3).

**Table 3:** Laboratory Values of Patients according to different NRS-2002 scores CRP: C-reactive protein  CAR: CRP to albumin ratio, NRS: Nutritional Risk Score

	NRS 1 or 2 (n:45)	NRS ≥ 3 (n:348)	P
	Med (IQR)	Med (IQR)	
Albumin (g/L)	31.15 (5.60)	28.15 (7.90)	<0.001
CRP (mg/L)	46.00 (70.11)	97.64 (106.64)	<0.001
CAR	1.47 (2.40)	2.40 (4.48)	<0.001

When the patients' laboratory values (albumin, CRP, CAR) were compared according to BMI, there was no significant difference in laboratory values according to BMI (p > 0.05).

According to the results of the correlation analysis between NRS-2002 and laboratory values of the patients, there was a statistically significant negative correlation between NRS-2002 and albumin level (p < 0.001). A positive low level statistically significant correlation was observed between NRS-2002 and CRP and CAR (p < 0.001), (Table 4).

**Table 4:** Results of Correlation Analysis between Patients' NRS-2002 Score and Laboratory Values CRP: C-reactive protein  CAR: CRP-to-albumin ratio, NRS: Nutritional Risk Score

		NRS-2002
Albumin	r	-0.179
	p	<0.001
CRP	r	0.219
	p	<0.001
CAR	r	0.232
	p	<0.001

Factors thought to be predictive of patients' NRS 2002 scores were included in the univariate logistic regression analysis. Age with p < 0.25 in univariate analysis and CAR variables were included in multivariate analysis. Accordingly, age and CAR were found to be effective in predicting those with NRS-2002 ≥ 3 (Table 5).

**Table 5:** Multivariate Logistic Regression Analysis to Determine Factors Affecting NRS 2002 Score in Patients Wald: test statistics, OR: odds ratio, CI: Confidence interval. Statistically significant p-values are in bold. CRP: C-reactive protein  CAR: CRP-to-albumin ratio

		Multivariate Binary Logistic Regression Analysis			
		Wald	p	OR	%95 CI for OR
Step 1	Age	4.394	0.036	1.027	(1.002-1.053)
	Gender (reference: male)	1.177	0.278	1.585	(0.690-3.641)
	CRP	0.012	0.913	0.999	(0.974-1.024)
	CAR	1.820	0.177	1.618	(0.804-3.256)
Step 2	Age	4.407	0.036	1.027	(1.002-1.053)
	Gender (reference: male)	1.179	0.277	1.585	(0.690-3.643)
	CAR	19.087	<0.001	1.559	(1.278-1.903)
Step 3	Age	4.671	0.031	1.027	(1.003-1.053)
	CAR	18.994	<0.001	1.561	(1.278-1.908)

In the ROC analysis, the area under the processing characteristic curve (AUC) for albumin, CRP, and CAR was calculated as 0.339, 0.757, and 0.759, respectively. Albumin, CRP, and CAR were found to be statistically significant (p < 0.05). When the cut-off value was accepted as 28.65 in terms of albumin level, the sensitivity was calculated as 53.7% and specificity as 75.0%. When the cut-off value for CRP level was accepted as 92.39, sensitivity was calculated as 52.7%, specificity as 93.3%. When the cut-off value for CAR was accepted as 3.26, the sensitivity was calculated as 50.4% and specificity as 93.3% (Table 6).

**Table 6:** ROC Curve Analysis Results for Laboratory Values in Patients AUC: area under the curve, PPV: positive predictive value, NPV: negative predictive value, LR+: positive likelihood ratio, LR-: negative likelihood ratio, CRP: C-reactive protein  CAR: CRP-to-albumin ratio

	Albumin (g/L)	CRP (mg/L)	CAR
AUC	0.339	0.757	0.759
AUC for 95% CI	(0.263-0.414)	(0.692-0.823)	(0.693-0.824)
P values	<0.001	<0.001	<0.001
Cut Off	28.65	92.39	3.26
Sensitivity	53.7%	52.7%	50.4%
Specificity	75.0%	93.3%	93.3%
PPV	94.4%	98.4%	98.3%
NPV	17%	20.4%	19.6%
LR+	2.15	7.87	7.52
LR-	0.62	0.51	0.53

## Discussion

In this study, 42.4% of the patients evaluated by the nutrition department were hospitalized for COPD. The majority of patients hospitalized for COPD and evaluated by the nutrition department were male. It was determined that nutritional therapy was initiated in almost all of the patients, regardless of BMI and NRS-2002. While it was determined that the nutritional treatment methods (TPN, EN, ONS) started were independent of BMI and NRS-2002, there was a negative relationship between NRS 2002 and albumin, and a positive relationship between CRP and CAR. The cut-off values of 28.65, 92.39, and 3.26 for albumin, CRP, and CAR, respectively, were significant in terms of determining that the patients were at nutritional risk. This study is particular in terms of evaluating the relationship between nutritional status and laboratory parameters in COPD patients.

The relationship between high morbidity and mortality in COPD and nutritional disorders cannot be ignored [3,4]. The high prevalence of malnutrition seen in patients with COPD also supports this situation [14]. Chronic inflammation in patients with COPD diagnosis distinguishes these patients from many patient groups [5]. Many chronic diseases associated with the inflammatory process are associated with increased protein catabolism [15]. Therefore, we think that the evaluation of nutritional status in COPD patients should be handled differently from other diseases. NRS-2002 is frequently used in the nutritional risk assessment of patients in clinical practice. It is stated that there is a nutritional risk in patients with NRS-2002 ≥ 3 and that it should be discussed in detail from a nutritional point of view [16]. However, in our study, the proportion of patients who did not need nutritional treatment and who were followed up on nutrition was significantly higher in the group with NRS-2002 one or two. In the group with low NRS-2002, 86.7% needed nutritional support. The high requirement for nutritional support in the group with low NRS 2002 may indicate that patients with COPD should be evaluated differently from a nutritional point of view. This high rate shows how important the clinician's observation and decision-making skills are in determining nutritional needs in patients with COPD.

BMI was used as a diagnostic criterion in most studies evaluating malnutrition in COPD patients and it has been reported that this index is associated with increased mortality and a high risk of acute exacerbations in patients with COPD [17]. In addition, studies indicate that low BMI is associated with sarcopenia, and sarcopenia is associated with malnutrition and a worse clinical course in patients with COPD [15,18]. In our study, it was observed that patients with COPD needed nutritional support independent of BMI. Although different BMI classifications and different BMI values ​​(Malnutrition = BMI < 18.5 kg/m2, < 20 kg/m2 or < 21 kg/m2) were used in the studies to define malnutrition, in our study, patients were evaluated in four groups according to BMI and the BMI < 18.5 kg/m2 was stated as underweight [19-21]. In the present study, nutritional support was needed not only in the underweight group, but also in 98% of the patients with normal BMI, 94% of those who were overweight, and 99% of those who were obese. Some studies state that BMI cannot distinguish between body parts and does not always reflect the patient's muscle mass [22]. Therefore, loss of muscle mass and malnutrition may occur in a patient who is defined as obese according to BMI. In addition, obese patients may not be able to get their daily calorie requirements due to decreased oral intake. Therefore, studies now focus on the low fat-free mass index (FFMI) in malnutrition assessment [23,24]. Only NRS-2002, FFMI, or BMI are not used in the diagnosis of malnutrition and sarcopenia in patients. Apart from these, nutritional risk assessment is performed using anthropometric measurements, blood tests such as serum albumin and cholesterol values, mini-nutritional assessment (MNA), and scoring systems such as Subjective Global Assessment (SGA) [22,25]. Therefore, studies in the literature indicate that standardized objective criteria are needed to diagnose malnutrition and sarcopenia in patients with COPD. In our study, we thought that CAR could be useful in determining the risk of malnutrition in patients with COPD, based on the inflammatory process in COPD and the negative effect of muscle mass loss on the prognosis in these patients. In our study, we investigated whether CAR is an effective parameter in nutritional assessment and evaluated its relationship with NRS-2002. A negative correlation was found between NRS 2002 and albumin, and a positive correlation between CRP and cut-off values ​​of 28.65 for albumin and 92.39 for CRP were determined. These cut-off values ​​may also be useful in terms of nutritional risk assessment in patients with COPD, but we think that CAR, where both values ​​are combined, can make a more effective assessment. We determined that the CAR value was significantly higher in those with NRS-2002 ≥ 3 and a cut-off value of 3.26 could be used for nutritional risk assessment. In addition, according to the regression analysis results in the present study, CAR can predict higher NRS - 2002.

Oral nutrition options should always be the first choice in patients who are at risk of malnutrition and need nutritional support [26]. In patients with a risk of malnutrition, ONS is recommended when dietary supplementation is not sufficient to meet nutritional goals [26]. It has been stated that dietary counseling and food supplementation are not sufficient to achieve nutritional goals, ONS provides higher protein and energy intake than dietary counseling, and improves the quality of life [26,27]. In the present study, it was observed that ONS was started in 76.3% of patients with COPD. However, it should not be forgotten that dietary counseling should be continued in patients who are started on ONS, and the complementary nature of ONS to the normal diet.

It is recommended to start EN if oral intake cannot be achieved for more than three days or if the energy requirement is expected to be less than half of the target for more than one week [26]. It is stated that TPN support is required in cases where EN cannot be provided or is contraindicated [26]. In the studies, we could not reach clear results on the rates of nutritional treatments applied especially to specific patient groups. In this study, while TPN was applied to 10.2% of the patients, EN was started to 10.9% of them.

This study has several limitations. First, it is a single-center and retrospective study. Due to the retrospective nature of the study, all patients with a diagnosis of COPD and who required hospitalization were evaluated, but no clear information on the severity of COPD disease could be obtained. In addition, it was observed that only NRS-2002 was used in nutritional assessment. Another limitation of our study is; After the nutritional support treatments were applied, data on the improvement of the nutritional status of the patients could not be obtained. Therefore, laboratory values could not be evaluated after nutritional support treatment. Although studies conducted in recent years have focused on the fat-free mass index (FFMI), FFMI calculations of the patients could not be made due to the retrospective nature of our study.

## Conclusions

In conclusion, the need for nutritional support is high in patients hospitalized with COPD. It is important to evaluate nutritional support requirements in these patients, regardless of NRS-2002 and BMI. In this respect, the clinician's observation and the decision are as valuable as the scoring that determines malnutrition. The oral route should be the primary choice in patients who require nutritional support, and the effectiveness of clinicians in nutritional assessment is important regardless of nutritional assessment. The cut-off values of 28.65 g/L and 92.39 mg/L for albumin and CRP, respectively, can be used to determine that patients with COPD are at risk in terms of nutrition. However, we think that the cut-off value of 3.26 for CAR, which combines CRP and albumin, can be used more effectively in nutritional risk assessment in patients with COPD. We think that prospective randomized controlled and multicenter studies will be guided in this regard.
